# William H. Dwinelle, A.M., M.D., D.D.S.

**Published:** 1896-04

**Authors:** 


### Obituary.

### WILLIAM H. DWINELLE, A.M., M.D., D.D.S.

THE death of this distinguished member of the dental profession occurred too late last month to secure adequate data of his life-work. We are indebted to his niece, Louise Fiske Bryson, M.D., of New York, for the following interesting account of his life:

"He was born July 22, 1819, and was the son of Louise Whipple and Judge Justin Dwinelle, of old New England stock, and of French and English ancestry.

"He was instrumental, with Dr. Chapin A. Harris, in establishing the first dental college in the world; he was also the associate editor of the first dental journal ever published.

"Dr. Dwinelle studied dentistry first with Dr. Douglass, of Albany, N. Y. He was always fascinated by surgery, and forty years ago performed operations upon the jaw for removal and resection similar to those that are considered triumphs of modern surgery.

"He made a European tour in 1851–52, visiting the World's Fair in London, and wrote numerous letters for publication concerning medicine and dentistry.

"A treatise on crystal gold was prepared by him in 1855; also papers, in the early fifties, on the use of the microscope in clinical medicine and dentistry, and, in addition, wrote important monographs on scientific subjects.

"An omnivorous reader, books and pictures were his chief delight. At one time he owned a valuable library and art collection.

"He was always the friend of aspiring and struggling artists, students, literary and professional men, aiding them by money and encouragement.

"For many years he was a member of the Union League Club, National Academy of Design, Jockey Club, Historical Society, St. Nicholas Society, County Medical Society, foreign associations, and New York Odontological Society, of which he was at one time president. He possessed a commanding figure and much personal charm. In his best days he was a brilliant after-dinner speaker. Always eloquent, it was said of him that, if interested in his subject, he could make people believe black was white.

"Through force of genius and industry he amassed a large fortune, that later, when disease overtook him, was dispersed in various hazardous speculations.

"He was unmarried. Of a family originally large, only a sister and brother survive him."

Dr. Dwinelle's work has been an intimate part of the history of dentistry for the past fifty years. The journals teem with his original ideas, and he was an authority upon all matters of practice during almost that entire period. The results of his labor, while original, were generally of that fugitive character not unusual with original minds, that it would require a prolonged research throughout the literature of the profession to give anything approaching a detailed account of it.

He was honored in his day and generation, and never failed to receive close and deferential attention whenever in later years he presented himself before the American Dental Association. Few of those present will fail to remember the scene before that body on one occasion, when Dr. Dwinelle stood upon his feet to discuss a matter before it, and was called to order by a member on parliamentary grounds. The answer of that meeting, refusing to entertain that objection, was a generous response and a cordial recognition of the services he had rendered the profession.

Still later, when, broken in health, crushed by circumstances that had financially wrecked him, he retired to the old homestead to pass the remainder of his life, it was stated in that same Association that Dr. Dwinelle needed help, who can forget the immediate and generous response? And it now remains for those who contributed to make Dr. Dwinelle's last days comfortable to feel that not only did they perform a gracious act, but they have laid up for themselves treasures that will count for something in the great summing up of good deeds.

Dr. Bryson has alluded to his charming conversational powers. Those who had the pleasure of meeting him in social intercourse can testify to the truth of this statement. His mind was stored with the products of the best literature of the past and present time, and, with a tenacious memory, he could bring treasures from this storehouse to brilliantly illuminate an hour's converse or an after-dinner address.

He was almost the last of that brilliant coterie of men who made dentistry. This may seem a strong assertion, in view of all that has been accomplished in later years, but in any work it is always the original efforts that direct the course of the future. It was fortunate that dentistry at a critical period secured the devoted services of men of learning, both in this country and abroad, —men capable of infusing the true educational spirit into those who were to succeed them.

We thus leave Dr. Dwinelle, the generous friend, an original worker, a brilliant companion, and a true professional colleague; for one by one the funereal bell is tolling the requiem of departed worth, and as the mourners' train passes on, let us who remain gather up the treasures these have left, that nothing be lost.

